# Phacoemulsification with Kahook Dual Blade goniotomy in eyes with medically treated glaucoma: analysis of surgical outcomes and success predictors

**DOI:** 10.5935/0004-2749.20220046

**Published:** 2025-08-21

**Authors:** Larissa Fouad Ibrahim, Anna Flavia Ribeiro Pereira, Larissa Alves de Oliveira Terenzi, Marcos Pereira Vianello, Syril Kumar Dorairaj, Tiago Santos Prata, Fabio Nishimura Kanadani

**Affiliations:** 1 Instituto de Olhos Ciências Médicas, Belo Horizonte, MG, Brazil; 2 Glaucoma Department, Instituto de Olhos Ciências Médicas, Belo Horizonte, MG, Brazil; 3 Mayo Clinic, Jacksonville, Florida, USA; 4 Universidade Federal de São Paulo, São Paulo, SP, Brazil

**Keywords:** Glaucoma, Glaucoma, open angle, Cataract, Phacoemulsification, Intraocular pressure, Goniotomy, Glaucoma, Glaucoma de ângulo aberto, Catarata, Facoemulsificação, Pressão intraocular, Goniotomia

## Abstract

**Purpose:**

The purpose of this study was to investigate the postoperative outcomes and evaluate the success predictors of phacoemulsification with Kahook Dual Blade goniotomy for cataract and glaucoma management in eyes with primary open-angle glaucoma.

**Methods:**

This was a retrospective, non-comparative; interventional case series in which all patients with primary open-angle glaucoma who underwent phacoemulsification with Kahook Dual Blade goniotomy between June 2018 and April 2019 were enrolled. All the participants had a minimum follow-up period of 6 months. Preoperative and postoperative intraocular pressure values (at 1, 3, and 6 months), number of antiglaucoma medications, best-corrected visual acuity, surgical complications, and any subsequent related events or procedures were recorded. A logistic regression analysis was performed to investigate the association between the different variables and surgical outcomes.**Results:** A total of 47 patients (57 eyes) were included (mean age, 70.5 ± 7 years). The mean intraocular pressure was reduced from 15.5 ± 4.2 mmHg to 12.2 ± 2.4 mmHg at the last follow-up visit (p<0.001). The mean number of antiglaucoma medications decreased significantly from 1.9 ± 1.0 to 0.6 ± 1.0 during the same period (p<0.001). On the basis of the predefined criterion (intraocular pressure reduction ≥20% and/or reduction ≥1 medication), the 6-month success rate was 86%. A higher preoperative intraocular pressure value (odds ratio [OR]= 2.01; p=0.016) and greater percentage of initial (30 days) intraocular pressure reduction (OR= 1.02; p=0.033) were sig nificantly associated with surgical success.

**Conclusion:**

Our findings suggest that phacoemulsification with Kahook Dual Blade goniotomy is an effective and safe alternative for cataract management in eyes with primary open-angle glaucoma that positively impacts intraocular pressure control and medication burden. Eyes with higher baseline intraocular pressure and a more pronounced initial response to the procedure appeared to present better outcomes at 6 months. Further studies are needed to evaluate the long-term efficacy and safety profile of the procedure.

## Abstract (pt)

**Objetivo:**

Investigar os resultados pós-operatórios e avaliar os preditores de sucesso da facoemulsificação combinada à goniotomia com o Kahook Dual Blade para o tratamento da catarata e do glaucoma em olhos com glaucoma primário de ângulo aberto.

**Métodos:**

Série de casos retrospectivos, não comparativos e intervencionistas, em que todos os pacientes com glaucoma primário de ângulo aberto submetidos ao procedimento de facoemulsificação combinada à goniotomia com o Kahook Dual Blade entre junho de 2018 e abril de 2019 foram inscritos. Todos os participantes tiveram um acompanhamento mínimo de 6 meses. Foram registrados os valores de pressão intraocular pré e pós-operatória (em 1, 3 e 6 meses), número de medicamentos antiglaucomatosos, melhor acuidade visual corrigida, complicações cirúrgicas e quaisquer eventos ou procedimentos subsequentes relacionados. A análise de regressão logística foi usada para investigar a associação entre diferentes variáveis e resultados cirúrgicos.

**Resultados:**

Um total de 57 olhos de 47 pacientes foram incluídos (média de idade, 70,5 ± 7 anos). A pressão intraocular média reduziu de 15,5 ± 4,2 mmHg para 12,2 ± 2,4 mmHg na última visita de acompanhamento (p<0,001). O número médio de medicamentos antiglaucomatosos diminuiu significativamente de 1,9 ± 1,0 para 0,6 ± 1,0 durante o mesmo período (p<0,001). Com base no critério predefinido (redução da pressão intraocular ≥20% e/ou redução de ≥1 medicamento), a taxa de sucesso em 6 meses foi de 86%. Um valor de pressão intraocular pré-operatório mais alto (OR= 2,01; p=0,016) e maior porcentagem de redução da pressão intraocular inicial (30 dias) (OR= 1,02; p=0,033) foram significativamente associados ao sucesso cirúrgico.

**Conclusão:**

Nossos resultados sugerem que o procedimento de facoemulsificação combinada à goniotomia com o Kahook Dual Blade é uma alternativa eficaz e segura para o manejo da catarata em olhos com glaucoma primário de ângulo aberto, impactando positivamente no controle da pressão intraocular e no número de medicamentos. Olhos com pressão intraocular basal mais alta e resposta inicial mais pronunciada ao procedimento parecem apresentar melhores resultados em 6 meses. Mais estudos são necessários para avaliar a eficácia em longo prazo e o perfil de segurança.

## INTRODUCTION

Glaucoma is a progressive chronic optic neuropathy characterized by typical alterations of the optical nerve head, retinal nerve fiber layer, and/or visual field^(1)^. It is most often accompanied by high intraocular pressure (IOP) that is considered within normal limits statistically. The treatment goal is to promote stabilization and delay or prevent the appearance of glaucomatous changes by reducing the IOP^(2-5)^. Oral or topical medications, and laser or surgical therapy are the main options to control IOP^(5-7)^. These treatments are aimed at decreasing aqueous humor production or increase its outflow^(5-9)^.

**Figure 1:**
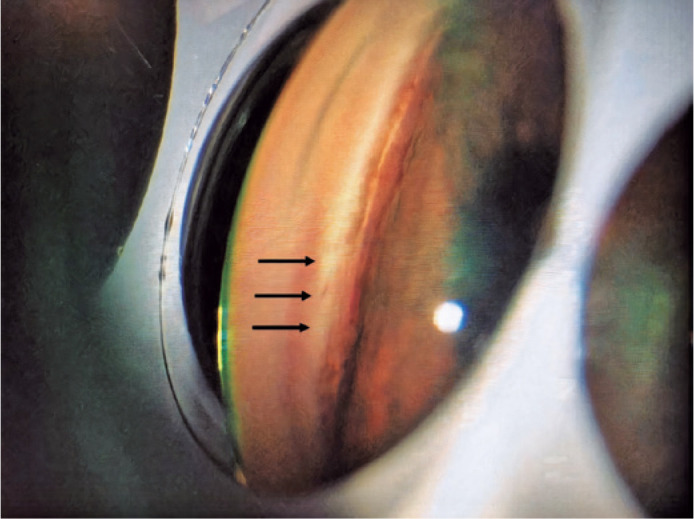
Gonioscopy image showing the KDB goniotomy site.

In the case of open-angle glaucoma (OAG), the trabecular meshwork (TM) is considered the site of greater resistance to the aqueous humor drainage^(10-13)^. The Kahook Dual Blade (KDB, New World Medical, Rancho Cucamonga, CA) is an ophthalmic knife that was initially marketed for use as a standalone treatment for TM removal in eyes that require a goniotomy^(9,14)^. The KDB device provides a minimally invasive removal of the TM strip through a small corneal incision. It is designed with a cone at the tip to allow smooth entry of the blade into Schlemm’s canal. Once properly supported in the channel, the device advances along the TM (Figure 1). The ramp at the distal end of the instrument lifts the TM tissue and guides it toward the blades on each side of the device, which cleanses the tissue to facilitate removal. By elevating TM and stretching it before cutting, the design allows a cleaner removal of the tissue and minimizes damage to adjacent structures^(14-17)^.

Published studies that investigated the efficacy and safety of KDB ranged from retrospective case series to prospective and comparative clinical trials^(6,10)^. However, only few studies have evaluated the most suitable patient profile for undergoing this surgical technique, especially when combined with cataract surgery. Knowledge of the possible success predictors in patients undergoing phacoemulsification combined with cataract extraction would likely facilitate the establishment of more-consistent protocols for indication of the procedure and follow-up. Therefore, in the present study, we aimed to investigate the postoperative outcomes and evaluate the success predictors of phacoemulsification with KDB goniotomy (PhacoKDB) for cataract management in eyes with primary OAG (POAG).

## METHODS

This was a retrospective, non-comparative, interventional case series in which all patients with POAG who underwent the PhacoKDB procedure between June 2018 and April 2019 were enrolled. All the procedures were performed at the Instituto de Olhos Ciências Médicas, Belo Horizonte, MG, Brazil (IOCM). All the patients had a minimum postoperative follow-up period of 6 months. The study followed the principles stipulated in the Declaration of Helsinki and was initiated after the approval of the IOCM ethics committee.

The inclusion criteria were patients with POAG or ocular hypertension, stable disease, mild or moderate functional damage, and symptomatic cataract. The patients were submitted to slit-lamp biomicroscopy, dilated fundoscopy, gonioscopy, IOP measurement using Goldmann applanation tonometry, and a visual acuity test (Snellen chart). The collected data included the patients’ demographic and ocular characteristics. The preoperative and postoperative IOP values (at 1, 3, and 6 months), number of antiglaucoma medications, best-corrected visual acuity, surgical complications, and any subsequent related events or procedures were recorded.

Surgical success was defined as follows: IOP reduction >20% in relation to the baseline IOP and/or reduction of at least 1 medication (if the postoperative IOP remained up to ±2 mmHg from the baseline IOP values). As possible predictors of success, we evaluated sex, age, the possible presence of hyphema, visual field mean deviation index, preoperative number of medications, preoperative IOP, and postoperative IOP change (%) at 1 month.

KDB goniotomy was performed after completion of the cataract surgery with intraocular lens implantation, in accordance with the manufacturer’s directions for use, with the assistance of a Swan-Jacob gonio prism. The TM was excised in the 4 to 5 o’clock position nasally. Postoperatively, 1% prednisolone acetate suspension, 0.5% moxifloxacin solution, and 0.5% ketorolac solution were prescribed. After 1 week, moxifloxacin therapy was discontinued, and the remaining medications were terminated 3 weeks later. The patients who received topical ocular hypotensive agents were advised to maintain their use, but the use of eye drops was suspended depending on the postoperative IOP measurements.

In the statistical analysis, numerical variables were presented as mean ± standard deviation, and categorical variables, as absolute and relative frequencies. In the patients whose both eyes were included in the analysis, to evaluate the demographic variables (information that did not vary according to the evaluated eye, such as sex and age), duplicated data were removed. For evaluating the specific variables in each eye, such as IOP, visual field mean deviation index, and number of medications, both eyes of each patient were considered independently. To evaluate the association between the categorical variables, the chi-square test was used. Numerical variables were submitted to the Shapiro-Wilk normality test. The preoperative and postoperative continuous variables were compared using a paired sample *t* test. The association between the possible success predictors and posto perative success was assessed using logistic regression modeling. The role of each variable is expressed in odds ratio and its 95% confidence interval. A significance level of 5% was used, and the analyses were performed using the R version 3.4.3 software.

## RESULTS

This study included data from 57 eyes of 47 patients. The profiles of the patients included in this study are shown in table 1. The mean patient age was 70.5 years (range, 53-86 years).

**Table 1:** Demographic and ocular characteristics of the study patients

Variables^*^	Patients (n=57 eyes)
Age (years)	70.5 ± 7.0
Sex (female/male)	28/19
Diagnoses (POAG/OH)	55/2
Visual field mean deviation index (dB)	-5.9 ± 6.0
Preoperative best-corrected visual acuity	0.6 ± 0.2

* Continuous data are expressed as mean ± standard deviation unless otherwise indicated. POAG= primary open-angle glaucoma; OH= ocular hypertension.

**Figure 2:**
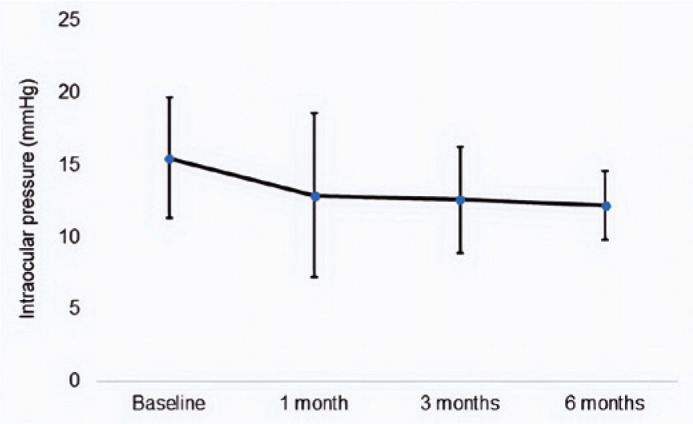
Mean intraocular pressure values at baseline and at each postoperative time point. The error bars indicate the standard deviation.

The mean IOP was significantly reduced from baseline to 6 months after operation (p<0.001), from 15.5 ± 4.2 mmHg to 12.2 ± 2.4 mmHg (Figure 2). The mean number of topical hypotensive medications also decreased significantly from baseline to 6 months after operation (p<0.001), from 1.9 ± 1.0 to 0.6 ± 1.0 at the last visit. The mean preoperative and postoperative best-corrected visual acuity values were 0.5 ± 0.2, and 0.9 ± 0.1 (Snellen chart), respectively, showing a significant improvement after the intervention (p=0.004).

On the basis of the predefined criterion (IOP reduction ≥20% and/or reduction ≥1 medication), the 6-month success rate was 86%. A higher preoperative IOP value (OR, 2.01; p=0.016) and greater percentage of initial (30 days) IOP change (OR, 1.02; p=0.033) were significantly associated with surgical success. Age, sex, and preoperative visual field mean deviation index, and number of glaucoma medications were not significant success predictors (Table 2).

**Table 2:** Factors associated with postoperative success of PhacoKDB

	OR	CI	p Value
Female sex	0.58	0.13-2.60	0.478
Age (for each year older), years	0.96	0.85-1.07	0.440
Presence of hyphema	0.48	0.11-2.19	0.347
Visual field MD index (for each dB of worsening)	1.06	0.92-1.24	0.403
Preoperative number of medications	1.73	0.80-3.76	0.166
Preoperative intraocular pressure, mmHg	2.01	1.14-3.75	0.016
Postoperative IOP change at 1 month, %	1.02	1.00-1.04	0.033

Phaco= phacoemulsification; KDB= Kahook Dual Blade; OR= odds ratio; CI= confidence interval; MD= mean deviation; IOP= intraocular pressure; dB= decibel.

Serious complications were not reported in any case. Of the 57 surgeries, 20 (35.1%) resulted in transient hy phema and 8% resulted in localized iridodialysis. Most of the cases of iridodialysis were mild (<2 hours) and probably secondary to a more anterior iris placement and/or a previous intraoperative anteriorization of the iris lens diaphragm.

## DISCUSSION

Our results corroborate the idea that PhacoKDB is a safe procedure and can be a good option for reducing the use of ocular hypotensive medications in patients with indications for cataract extraction. To the best of our knowledge, this is the first study conducted in Brazil on this topic.

A mean IOP reduction of 3.3 mmHg was observed after 6 months in our study, which was slightly lower than that in the study of Greenwood et al, who reported a mean 6-month postoperative reduction of 4.6 mmHg^(10)^. However, the referred research included patients with other diagnoses such as angle-closure, pigmentary, pseudoexfoliative, and normal-tension glaucoma, in addition to presenting high mean IOPs at baseline.

Six months after the procedure, 86% of the patients met the success criteria. This result is comparable with and even more favorable than that in the study by Sieck et al., who reported a success rate of 74.1% at 6-month postoperative follow-up^(6)^.

Few data have been reported in the literature regarding the evaluation of success predictors for PhacoKDB. In the article by Sieck et al., no association was indicated between postoperative success and sex, age, number of preoperative medications, and baseline IOP, with data from 12 months after surgery^(6)^. These results are similar to our study results, except for the IOP at baseline, in which we observed a significant association between postoperative success and higher IOP. This association seems to make sense owing to the flooring effect of any Schlemm’s channel-based procedures, where IOP lowering cannot be achieved beyond the level of episcleral venous pressure^(18)^. Another association found in this study was between treatment success and IOP reduction 1 month after surgery. This should be used to determine between an expectant and interventionist conduct in a shorter time.

Considering that this study did not include a control group with POAG treated with phacoemulsification surgery alone, we searched the literature for comparative data. Most of the available studies performed a retrospective analysis. In a recent research by Majstruk et al., 70 eyes of 40 patients with POAG who underwent cataract surgery by phacoemulsification were retrospectively evaluated. After 1 year, IOP decreased by a mean 1.15 ± 3 mmHg (6.8% ± 18.1%), and the number of glaucoma medications was almost unchanged, with a difference of only -0.1 ± 0.43^(19)^. Another study conducted by Slabaugh et al. evaluated 157 eyes with OAG controlled with medication. After 1 year of follow-up, the IOP was reduced by a mean of 1.8 ± 3.1 mmHg, but no significant change in the number of medications was observed after cataract surgery^(20)^. In rigorous studies, the mean long-term changes in IOP value ranged from 1.5 and 2 mmHg. However, whether this percentage reduction in IOP is clinically meaningful remains unclear^(21)^, also considering that no statistically significant reduction in the number of eye drops was found.

In terms of postoperative adverse effects, PhacoKDB seems to be a safe procedure^(19)^. Transient hyphema was observed in 20 eyes (35%). However, all the cases presented spontaneous resolution in the first postoperative month without the need for further intervention. This is consistent with the results of other studies^(6,10)^ in that the most noticeable adverse events after the procedure were pain, ocular discomfort, iridodialysis (1.4%-1.9%), and tear of Descemet’s membrane (1.4%-1.9%)^(10)^.

The study has some limitations due to its retrospective nature. Though well experienced, three different surgeons performed the procedure, and the number of participants was limited because the procedure was introduced at the hospital just 1 year before the study. Another limitation is the absence of a control group, as our focus was on evaluating success predictors.

Regarding the nomenclature, we believe that the KDB procedure should be considered an ab interno trabeculectomy instead of a goniotomy, as during the procedure, part of the trabecular membrane is not only cut but excised (Figure 1). Our findings suggest that the PhacoKDB procedure is an effective and safe alternative for cataract management in controlled mild to moderate glaucoma with indications for phacoemulsification that positively impacts IOP control and medication burden. Eyes with a higher baseline IOP and more pronounced initial response to the procedure appeared to present better outcomes at 6 months after operation. We highlight that we did not perform a standalone procedure because all the patients had a well-controlled mild to moderate glaucoma with indication for phacoemulsification due to cataract. This criterion is different from those in most published studies.
